# It is the time to change the paradigms of pregnant and breastfeeding women in clinical research!

**DOI:** 10.3389/fphar.2023.1113557

**Published:** 2023-02-23

**Authors:** Liberata Sportiello, Annalisa Capuano

**Affiliations:** ^1^ Campania Regional Centre for Pharmacovigilance and Pharmacoepidemiology, University of Campania “Luigi Vanvitelli”, Naples, Italy; ^2^ Department of Experimental Medicine, University of Campania “Luigi Vanvitelli”, Naples, Italy

**Keywords:** pregnancy, breastfeeding, women, vulnerability, clinical research, paradigm

## 1 Introduction

Women’s physiological changes in pregnancy and breastfeeding may alter the pharmacokinetics and the pharmacodynamics of many drugs, resulting in potential risks to both the mother and fetus/child (Pinheiro and Stika, 2020). Moreover, medicinal products may have a different impact according to the stage of pregnancy. For this reason, pregnant and breastfeeding women are historically considered a “susceptible population.” Based on their supposed susceptibility, it is well known that women in pregnancy and breastfeeding women are often excluded from clinical research and the general opinion is that this exclusion must be justified unless there are persuasive scientific explanations for their inclusion (van der Graaf et al., 2018). Moreover, when women of childbearing age are enrolled in clinical trials, many study protocols imposed the use of contraceptive methods during their trial participation, while this approach does not apply when men are the study participants.

What are the consequences of the historical approach of excluding pregnant and breastfeeding women from clinical trials? Although the lesson learned from thalidomide 60 years ago prompted the birth of modern pharmacovigilance and has unquestionably avoided further tragedies, the highly preemptive position in excluding these women from clinical trials could force individual woman and healthcare professionals in deciding alone on the assumption of drugs and exposure to potential risks during pregnancy and breastfeeding (Schonfeld, 2013). It is very relevant to minimize the maternal, fetal, and neonatal risks during a pharmacological treatment, but this is possible only if there is enough information from clinical trials on its safety profile during pregnancy and breastfeeding and clear indications on how to proceed. However, both pregnant and lactating women and healthcare professionals often do not have access to this type of information. Thus, we need a new strategy outline to better protect pregnant and lactating women using medicines.

This means that it is essential to move toward involving more pregnant and breastfeeding women in drug and vaccine clinical trials in order to collect, assess, and communicate in a more appropriate manner the data on medicine safety in pregnancy and breastfeeding. Therefore, the purpose of this article is to discuss the importance of the changes to be made in the paradigms of pregnant and breastfeeding women in clinical research.

### 1.1 Disproof of the historical approach and ethical considerations

The historical approach by regulators, medicine developers, marketing authorization holders, healthcare professionals, academics, and patients is to exclude the women in pregnancy and breastfeeding from clinical research because they are considered a “vulnerable population.” Vulnerability is a broad and complex concept, in which the medical and non-medical, social, and other risk factors play an important role (Scheele et al., 2020). Specifically, in clinical research, a vulnerable group or person is defined by the compromised ability to protect its interests and provide informed consent**.** However, although in pregnancy and breastfeeding conditions, these women have the same ability as men to decide on their participation in clinical trials. On the contrary, the physiological characteristics are different between women and men, and they may respond differently to treatments. This diversity further underlines the need in enrolling women, even if in pregnancy and breastfeeding, as participants in clinical research because it is not possible to generalize the data from those of men (Committee on Ethics, 2015).

In the last few years, it has been reported in the literature that several authors dealt with this issue and converging opinions have emerged in destroying the myth of vulnerability of pregnant and breastfeeding women. van der Zande et al. (2017) highlighted that the classification of these women as a “vulnerable population,” which should justify their exclusion from clinical trials, is deeply problematic and conflicting in the context of research. Unlike more diffuse opinions in considering pregnant and breastfeeding women as vulnerable, van der Zande et al. reported that if a woman is pregnant, she can offer valid consent or refusal to participate in a research. The only reason for a potential condition of vulnerability is that pregnant women are exposed to higher risks due to a lack of scientific knowledge. Additionally, Krubiner et al. also commented that the paradox of the exclusion of pregnant women from clinical trials does not protect them from potential risks but exposes them and their offspring to greater risks (Krubiner et al., 2017). In clinical practice, a pregnant woman receiving a drug for which there is limited data on its potential teratogenicity could be increasingly exposed to higher risks.

Toby Schonfeld’s viewpoint was even more drastic. In his opinion, the consideration that pregnant women are vulnerable subjects involves insidious perils due to the protective approach toward them. The main peril is not only to hinder essential research on pregnant women and their offspring but also negatively impact the inclusion of all women in clinical research (Schonfeld, 2013). van der Graaf et al. (2018) proposed an integrated scientific and ethical approach for the inclusion of pregnant and breastfeeding women in clinical trials. Fair inclusion of pregnant women means that eligible pregnant women are not excluded purely for being pregnant. Moreover, pregnant women should receive more attention and the research interests on them should be a priority. The common emerging opinion is that pregnant women in clinical trials should be redefined as a “scientifically complex” rather than a “vulnerable” population because of the more scientific efforts required (Farrell et al., 2020). This is the exact opposite of what happens because the outcomes of pregnancy were often not investigated and reported consistently. A recent systematic review found that clinical trials in pregnant women with diabetes, HIV infection, and hypertension did not report or only partially reported many essential offspring data (Aurich et al., 2020). Specifically, the number of births was frequently not reported, while congenital malformations, fetal losses, and neonatal deaths were often not reported with enough detail. Regulatory guidelines for the management and publication of safety data in clinical trials also regard data on the offspring and any potential effect of *in utero* exposure. However, there is uncertainty on what data should be collected in clinical trials and how they should be reported.

However, the question on what do pregnant women think about their participation in clinical trials rises. Jaffe et al. (2020) conducted an interview-based study with pregnant women, getting information on their opinions on several hypothetical Zika virus vaccine research scenarios and investigating their decision-making processes for participation. Women’s decision-making processes were based on the available evidence on vaccine safety profiles in pregnant animals and non-pregnant humans, the potential risks specifically related to the fetus or baby, and the trust in the person who recommended vaccine research participation. More recently, another study explored 258 pregnant women’s experiences and perceptions on maternal vaccination and maternal vaccine trials in France, Germany, Italy, Spain, and the United Kingdom (Karafillakis et al., 2021). Their findings showed that pregnant women had low awareness about maternal vaccination, underlying the fact that healthcare professionals did not recommend them getting vaccinated. Some women used the internet or social media to get information, particularly in France and the UK. About maternal vaccine trials, most pregnant women strongly rejected the involvement in trials because they considered it an unnecessary risk in pregnancy. Only few women had a positive opinion about taking part in trials in order to contribute to the progress of science or to benefit pregnant women in the future.

### 1.2 Need for more data on pregnancy and breastfeeding

Women commonly use medicines during gestation or breastfeeding. The use of prescribed and over-the-counter medications in these conditions has progressively increased over the last decades (Stultz et al., 2007; Lupattelli et al., 2014; Ayad and Costantine, 2015)**.** Much of drug exposure occurs accidentally because more than half of the pregnancies are unplanned. In addition to drug exposure before pregnancy detection, many maternal conditions, both acute (such as infections or pregnancy complications) and chronic (such as hypertension, asthma, diabetes mellitus, infections, or severe psychiatric disorders), may require pharmacological treatments after pregnancy is confirmed (Bérard et al., 2019; Office of the Surgeon General, 2020). Moreover, several diseases complicating pregnancy and breastfeeding are rising in prevalence, secondary to advanced maternal age (Halpern et al., 2019). However, most of these women do not know potential risks of the used medicines mainly related to the fetus/baby besides themselves (de Waard et al., 2019). A lot of evidence gaps exist in the use of many drugs in these special conditions of women’s life (Jaffe et al., 2020; Karafillakis et al., 2021)**.**

The evidence on the safety profile of medicines starts from preclinical research. The translation of preclinical data in clinical outcomes is not always consequential, and also, in the past, a single adverse effect in preclinical studies induced a preventive position, suggesting to avoid the use of the drug or reduce its doses in pregnant and breastfeeding women rather than to investigate its clinical risk (Nooney et al., 2021). To date, many drug manufacturers use innovative non-clinical methods, such as developmental and reproductive toxicology (DART) studies, physiologically based pharmacokinetic (PBPK) modeling, and *in vitro*-to-*in vivo* extrapolation modeling. These types of studies are indicative of potential risks in pregnant and lactating women and should be used mandatorily by regulatory agencies.

Regarding clinical research, the traditional approach did not generate robust data on medicine safety in pregnancy and breastfeeding, especially due to the uncertain and biased preclinical data and the preconceived exclusion of these women in clinical trials. Pregnant and breastfeeding women in clinical trials should be considered a scientifically and ethically complex population because they require more efforts to generate safety data. An issue regards the evaluation of the benefit/risk profile associated with the mother and fetus separately. Clinical studies can be conducted in order to benefit the pregnant woman and her fetus (e.g., a trial finalizing to evaluate appropriate tocolysis for prevention of preterm birth or drugs for the treatment of gestational diabetes) and non-pregnancy-related studies that may benefit a woman during her pregnancy, taking into account that pregnancies are increasingly occurring in women with older age or complex medical problems (Committee on Ethics, 2015). Moreover, an over-emphasis on potential harm seems to be given to the fetus without weighing the potential benefit to the pregnant women’s health and wellbeing. The evaluation should start with the severity and impact of the mother’s disease. Moreover, more thorough and appropriate benefit/risk considerations for the mother and the fetus/baby should be performed, taking into account whether the mother is treated or not. However, as suggested by Nooney et al. (2021), the basic principle in pharmacological research is the statement “*The health of the child begins with the health of the mother”* because maternal and fetal/neonatal risks are deeply associated. Moreover, the enrollment of pregnant and lactating women in clinical research requires a balance between the benefit/risk profile for both the mother and fetus in comparison with getting information on these special conditions. The evaluation of the benefit/risk ratio is yet more difficult if we consider a delay between the *in utero* exposure or during breastfeeding and the onset of an adverse outcome (e.g., birth defects, child developmental delay, etc.).

Moreover, regardless of clinical trial outcomes, the specific information should be collected in these subpopulations during a clinical trial. In particular, relevant data comprise the trimester and the course of pregnancy, pregnancy/labor/delivery complications, termination of pregnancy, and the risk of abortion for the mother, while congenital, familial, and genetic disorders, and fetal and neonatal disorders for the fetus/child, besides specific pregnancy-related outcomes predefined in the protocols. Moreover, these data should be reported in every scientific report or publications by medicine sponsors/manufacturers also as additional material.

In the post-marketing period, pharmacovigilance activities are fundamental for the monitoring of the tolerability profile of all medicines (Rafaniello et al., 2016; Scavone et al., 2016; Sessa et al., 2016). Several data sources are available in order to collect data on medicine safety, such as spontaneous adverse drug reaction systems (although it has well-known limitations), observational studies, and patient registries. However, also the evidence emerging from these data sources was not used appropriately in order to generate suggestions and indications for a careful decision-making process by regulators, drug manufacturers, pregnant and lactating women, and healthcare professionals (European Medicines Agency (EMA), 2020). A useful strategy for both pregnant and breastfeeding women and the fetus/baby could be the generation of global data using innovative methods (e.g., hybrid approach linking primary and secondary data, human breast milk studies/biobank, and artificial intelligence) in both pre- and post-marketing periods (European Medicines Agency (EMA), 2020).

### 1.3 Regulatory strategies and approaches in Europe

In Europe, few medicines have been proven to be teratogenic in humans as many as few medicines are clearly approved for pregnancy and breastfeeding because of the limited understanding of benefits and risks to the mother and fetus/child. Together with the United States Food and Drug Administration (US-FDA), the European Medicines Agency (EMA) is one of the world leaders in the field of overall medicine management and in pharmacovigilance. These regulatory agencies and many scientists are making great strides to improve knowledge of the rational use of medicines in the pregnant and breastfeeding populations.

In 2005, the EMA first approached this issue by releasing a guideline on the exposure to medicinal products during pregnancy, with the need for post-authorization data (European Medicines Agency (EMA), 2005). Specifically, this guideline provided indications on how to monitor accidental or intended exposure to medicinal products during pregnancy and specific requirements for reporting data and adverse outcomes of pregnancy exposure. In 2019, the EMA proposed another guideline with the aim to provide guidance to marketing authorization applicants/holders, competent authorities of member states, and the Agency for facilitating appropriate pharmacovigilance for medicinal products that may be used in pregnant or breastfeeding women (European Medicines Agency (EMA), 2019). In 2019, the EMA took part in the launch of the ongoing IMI-ConcePTION project, funded by a private public partnership between the Agency, public health organizations, drug manufacturers, and academia. The ConcePTION project is designing and building a lasting ecosystem of data collections, methods, people, and infrastructure that allow the generation and dissemination of evidence on medicine safety in pregnancy and lactation (Innovative Medicines Initiative, 2022). In early 2020, the EMA even launched a workshop to discuss how to implement the strategy outline to better obtain and communicate the evidence on medicine safety in pregnant and breastfeeding women, provide it appropriately, and enable decision-making on their medical treatment (European Medicines Agency, 2020a). The common opinion emerged from this workshop was also to collect the evidence for pregnant and breastfeeding women separately because they are two different subpopulations. This initiative was also included among the aims of EMA Regulatory Science to 2025, which underlined that this strategy should include considerations regarding PK/PD modeling, epigenetics, reproductive toxicity studies, clinical trial design, and post-authorization follow-up methods (European Medicines Agency, 2020b). In 2021, regulatory agencies such as the FDA, EMA, and Medicines and Healthcare products Regulatory Agency (MHRA, United Kingdom) published an article in which they discussed their shared approach to gain evidence on medicine safety for pregnant and breastfeeding women (Nooney et al., 2021).

In addition to the new ongoing initiatives, another approach of the EMA is to use better the existing methods, such as the Periodic Safety Update Report (PSUR) of medicines, EudraVigilance analyses, and translation into good risk minimization measures. In 2022, the EMA consulted the public for a new guideline (European Medicines Agency, 2022), which defines the elements of a pregnancy prevention programme (PPP) (when a product shows potential for a teratogenic effect or adverse effect on the (neuro)development of the child through *in utero* exposure) or what other risk minimization measures are considered appropriate to avoid adverse pregnancy outcomes due to the use of medicines and to preserve the health of both the mother and the child.

### 1.4 COVID-19 experience: What do we learn from it?

Currently, the issue about the inclusion of pregnant and breastfeeding women in clinical research was extremely debated, also considering the experience gained in the coronavirus disease 2019 (COVID-19) period (Whitehead and Walker, 2020). The COVID-19 pandemic has offered a unique opportunity of appropriately reconsidering pregnant and breastfeeding women in clinical research during infectious disease outbreaks, but it was not suitably seized. Smith et al. found that among 927 COVID-19-related trials in the World Health Organization International Clinical Trials Registry, 52% explicitly excluded pregnancy, 46% failed to address pregnancy, and only 1.7% was pregnancy-specific (Smith et al., 2020). Also, Kons et al. analyzed the rates of excluded pregnant and breastfeeding women in COVID-19 vaccine and treatment clinical trials, using the U.S. National Library of Medicine database (Clinicaltrials.gov). Here, 97.8% of 90 vaccine trials excluded pregnant women, and 81.1% excluded lactating women. Of the 495 COVID-19 treatment trials, 70.7% and 54.3% excluded pregnant and lactating women, respectively (Kons et al., 2022). Considering the high risks associated with SARS-CoV-2 infection, the pandemic condition has also imposed the need for pregnant and breastfeeding women to be vaccinated, although there was a lack of evidence from clinical trials. Only later, the data are emerged and are emerging again on the safety of COVID-19 vaccination during pregnancy and breastfeeding (Shimabukuro et al., 2021; Mascolo et al., 2022). Given the stark disparities that arise during COVID-19, the exclusion of pregnant and breastfeeding women from clinical studies as a blanket policy created inequities among populations.

However, in early 2020, regulatory agencies such as the EMA made a great deal of effort launching the CONSIGN study (European Medicines Agency, 2021). This ongoing European project aims to evaluate existing data sources (e.g., electronic health records and hospital data) and cohorts of pregnant women to provide information on the impact of COVID-19 and its treatments in different trimesters of pregnancy. The goal is to address decision-making about vaccine indications, vaccination policies, and treatment options for COVID-19 in pregnant women.

Moreover, during the COVID-19 pandemic, a high percentage of pregnant and breastfeeding women took medications not related to COVID-19 management. Ceulemans et al. (2022) underlined a highly prevalent medicine use by performing an anonymous web survey in several countries (Ireland, Norway, Switzerland, the Netherlands, and the United Kingdom) during the first pandemic wave. In particular, at least one drug has been used by about 60% of women, and daily and occasional assumption was reported by 34% and 42% of pregnant women and 29% and 44% of breastfeeding women, respectively. The most used medications were those acting on the nervous system, the respiratory system, the alimentary tract/metabolism, and the musculoskeletal system.

Despite the high medicine use during pregnancy and breastfeeding in the COVID-19 pandemic, these women were not inclined to expose themselves to drugs/vaccines during a clinical trial. In fact, in an interview-based study conducted to assess the acceptability of participating in COVID-19 clinical trials among pregnant women in Spain (Marbán-Castro et al., 2021), these women reported that they had received limited information, especially on the potential relation between pregnancy and the severe phase of the disease, which caused uncertainties and emotional suffering. In general, they had no propensity to participate in both COVID-19 and other drug clinical trials. Healthcare providers suggested the participation of pregnant women’s relatives during the recruitment visit of the clinical trial to support their decision.

## 2 Discussion

In this opinion article, we discussed the urgent need to move toward changes in the paradigms of pregnant and breastfeeding women in clinical research, disproving the historical approach in excluding these women from clinical trials. To the best of our knowledge, in the last few years, many authors debated on this issue and proposed a reconsideration of women in pregnancy and breastfeeding in clinical studies. Each year, hundreds of women confront illnesses, often significant and serious, while pregnant or breastfeeding, but information about how to treat these conditions is widely limited. Few studies are designed to address health concerns and questions relevant to pregnant and lactating women, and this results in a lack of evidence to guide healthcare professionals, treatment policies, and decisions. Therefore, there is women’s hesitancy to receive drugs or to get vaccinated during pregnancy and breastfeeding.

Over the last few years, many efforts have been made in order to encourage the inclusion of underrepresented populations, such as women and children, in clinical research. Women of childbearing age should have the same opportunity as males in deciding on their trial participation. Despite this consideration, pregnant and breastfeeding women remain an excluded population. The health needs of pregnant and breastfeeding women should be addressed taking into account the complexities of scientific and ethical aspects in clinical research. Moreover, pregnant and lactating women should be encouraged to participate in a clinical trial to increase their confidence for participant inclusion, such as better communicating preclinical evaluation results. The general unwillingness to include pregnant and breastfeeding women in clinical research was tangible also in COVID-19 experience. Although these women were at higher risk for severe illness and hospitalization or more stillbirths and preterm births (Khalil et al., 2020), they were not included in clinical trials for vaccines and treatments for COVID-19.

Thus, in the era in which the research is focalizing the attention on gender differences, we believe that each pre-clinical and clinical study should also be designed and assessed for the pregnancy and breastfeeding specific outcomes, regardless of trial outcomes. Indeed, in last few years, the regulatory agencies started a constructive approach and international cooperation between the different stakeholders (other regulators, industry, healthcare professionals, and patients) to better collect, evaluate, and communicate data on the benefit/risk ratio of medicines in pregnant and breastfeeding women. Each step of medicine development should be reevaluated, considering pregnancy and breastfeeding. A relevant aim in this field is the need to establish the criteria for conducting clinical trials which also involved pregnant and breastfeeding women. Incorporating their priorities into the trial design may facilitate their participation and generation of evidence for this important subpopulation. The revolutionary approach in reforming human research guidelines with a greater participation of pregnant and breastfeeding women represents the new paradigm which should allow shifting from protecting these populations from research toward protecting these populations through research. Moreover, because research on pregnant and breastfeeding women requires thoughtful study design, researchers should provide key items in study protocols to establish appropriately potential risks for the mother and her offspring so that pregnant and breastfeeding women and healthcare professionals can decide on the best treatment option.

We justify a reduced involvement of pregnant and breastfeeding women in the early phases of drug development (when there are low morbidity conditions) because little information is available on its effects, but this preventive approach should not be allowed in the later phases or in serious conditions (e.g., seizure or cardiac disorders).

To date, there is a breath of changes at the global level, but for the moment, these changes are still yet to be implemented. Therefore, in the early future, we trust in the substantial inclusion of pregnant and breastfeeding women in clinical studies and the development of robust electronic data collection and analysis globally. This should be brought to a new “approved” role of pregnant and breastfeeding women in the field of research.
